# International Importation Risk Estimation of SARS-CoV-2 Omicron Variant with Incomplete Mobility Data

**DOI:** 10.1155/2023/5046932

**Published:** 2023-09-14

**Authors:** Yan Zhu, Yuan Bai, Mingda Xu, Lin Wang, Thomas K. T. Li, Zhanwei Du, Yuexuan Wang

**Affiliations:** ^1^QICI Quantum Information and Computation Initiative, Department of Computer Science, The University of Hong Kong, Pokfulam Road, Hong Kong, China; ^2^WHO Collaborating Center for Infectious Disease Epidemiology and Control, School of Public Health, LKS Faculty of Medicine, Hong Kong Special Administrative Region, The University of Hong Kong, Hong Kong, China; ^3^Laboratory of Data Discovery for Health Limited, Hong Kong Science Park, Hong Kong Special Administrative Region, Hong Kong, China; ^4^Department of Genetics, University of Cambridge, Cambridge CB2 3EH, UK; ^5^Department of Obstetrics and Gynecology, Queen Mary Hospital, The University of Hong Kong, Hong Kong, China; ^6^AI Technology Lab, Department of Computer Science, The University of Hong Kong, Pokfulam Road, Hong Kong, China; ^7^College of Computer Science and Technology, Zhejiang University, Hangzhou, Zhejiang, China

## Abstract

A novel Omicron subvariant named BQ.1 emerged in Nigeria in July 2022 and has since become a dominant strain, causing a significant number of repeated infections even in countries with high-vaccination rates. Due to the high flow of people between Western Africa and other non-African countries, there is a high risk of Omicron BQ.1 being introduced to other countries from Western Africa. In this context, we developed a model based on deep neural networks to estimate the probability that the Omicron BQ.1 introduced to other countries from Western Africa based on the incomplete population mobility data from Western Africa to other non-African countries. Our study found that the highest risk was in France and Spain during the study period, while the importation risk of other 13 non-African countries including Canada and the United States is also high. Our approach sheds light on how deep learning techniques can assist in the development of public health policies, and it has the potential to be extended to other types of viruses.

## 1. Introduction

On July 10, 2022, a novel Omicron subvariant named BQ.1 emerged in Nigeria, according to Omicron genome sequence data shared via GISAID [1]. This Omicron subvariant has become a dominant strain rapidly since mid-August 2022 in Nigeria, a sublineage of BA.5 [2, 3]. Omicron BQ.1 (including its sublineages) causes a massive number of repeated infections, even in countries with high-vaccination rates due to its immune escape advantage over other Omicron subvariants [4]. In the 2 months since the first case was detected, Omicron BQ.1 has been detected in at least 10 countries and caused about 2,000 infected cases [2]. Considering the potential health burden that Omicron BQ.1 may cause, estimating the importation risk of Omicron BQ.1 may inform public health policymakers to adjust control measures before the virus spreads further at its destination.

Due to many daily flights between Western Africa and other non-African countries, a large flow of people make the risk of Omicron BQ.1 being introduced to other non-African countries from Western Africa very high. A prior study by Bai et al. [5] utilized flight data along with mobility data from Facebook and OpenSky [6] to estimate the global risk of Omicron variant importations that originated in South Africa in November 2021. The authors estimated the unreported mobility flows between the two countries via the daily number of flights and the proportion of aircraft seats occupied. Especially mobility flows with less than 1,000 daily users will not be reported by Facebook. The previous study made an assumption of a fixed ratio to represent the proportion of occupied aircraft seats, which may have had an impact on the accuracy of importation risk estimation. This simple assumption could particularly affect the estimation of the probability of multiple infected cases arriving at a destination, potentially leading to less precise results. In this work, we proposed a model based on deep neural network to estimate the importation risk of the Omicron BQ.1 variant, which is able to yield accurate risk estimation from incomplete mobility data. By harnessing the capabilities of deep neural networks, the proposed model accurately estimates the proportion of occupied aircraft seats for each flight between two countries, thereby improving the precision of the risk estimation. This advancement in estimation techniques sets our research apart from previous studies and contributes to the field by addressing the limitations of existing approaches. We believe that our model represents a significant step forward in accurately estimating the importation risk of the Omicron BQ.1 variant, providing valuable insights for public health decision-makers.

## 2. Materials and Methods

### 2.1. Data

To estimate the daily number of travelers from Western Africa to other non-African countries, we analyzed the movement map provided by Facebook [7], describing the number of Facebook users who moved from one country to another daily. Note that Facebook reports the mobility flows between two countries only when at least 1,000 daily Facebook users travel between these two countries in the raw dataset, so only 261 intercountry links are considered from July 10, 2022 to September 30, 2022. To fill in missing data, we further analyzed the flight data provided by OpenSky [6] from July 10, 2022 to Sep 30, 2022. During the analysis, we also exploited information about different countries such as location, gross domestic product (GDP) per capita, and a series of indexes reflecting the government policy of the different countries. To correct possible geographic bias existing in the Facebook data, we also adopted the data indicating the proportion of population using Facebook in the different countries.

Based on the daily number of travelers estimated above, we further estimated the risk of importation from Western Africa to non-African countries. In our study, our primary focus was on 15 countries located in Western Africa, including Nigeria, along with 14 other countries: Morocco, Algeria, Tunisia, Senegal, Gambia, Cabo Verde, Cameroon, Benin, Burkina Faso, Chad, Ghana, Guinea, Guinea-Bissau, and Niger. The selection of these countries was based on two key criteria. First, we considered their proximity to Nigeria, ensuring that the distance from each country to Nigeria was less than 5,000 km. This criterion was essential as it allowed us to concentrate on neighboring countries with potential high-mobility flows. Second, we took into account the availability of sufficient flight data between these countries and other regions. Specifically, we ensured that each country had at least one flight that connected it to destinations outside the Western Africa. This criterion enabled us to analyze meaningful mobility patterns and potential importation risks associated with these countries. In our analysis, we aggregated Omicron BQ.1 (Omicron BQ.1 and its sublineages, such as BQ.1.1 and BQ.1.2). Given the geographical proximity and the unavailability of precise prevalence data of Omicron BQ.1 for other countries, we made the assumption that the prevalence of Omicron BQ.1 in Western Africa from July 10, 2022 to September 30, 2022, closely mirrored that in Nigeria, where Omicron BQ.1 was first detected [2]. This approximation allowed us to account for the potential spread of the variant during that time frame despite the data limitations.

### 2.2. Estimating Missing Daily Mobility Data from Western Africa to Other Non-African Countries by Deep Neural Network

**Figure 1:**
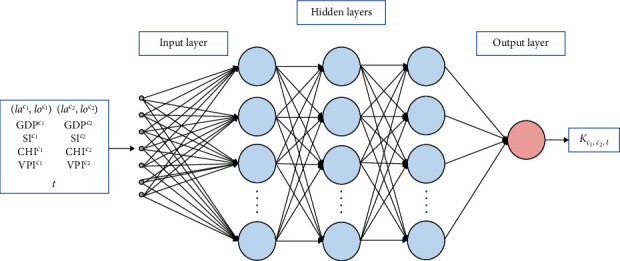
Structure of the neural network *f*_*θ*_ used for predicting *κ*_*c*_1_,*c*_2_,*t*_. It consists of three parts. The input layer accepts the time *t* and the information in two countries *c*_1_ and *c*_2_ including latitude and longitude, GDP per capita and a series of indexes reflecting thegovernment policy of *c*_1_ and *c*_2_. Specifically, SI, CHI, and VPI represent the stringency index,the containment- and -health- index, and the vaccination policy index, respectively. Thehidden layers are composed of multiple computing nodes with some trainable parameters *θ* and the output layer yields the predicted *κ*_*c*_1_,*c*_2_,*t*_.

Table 1 presents our notation and the corresponding parameter values. To estimate the missing data in Facebook movement map, we first built a deep neural network model [17] to predict *κ*_*c*_1_,*c*_2_,*t*_, which denotes the proportion of seats on flights from country *c*_1_ to country *c*_2_ that are occupied at time point *t*. The structure of the neural network is shown in Figure 1. This neural network can be regarded as a function mapping from the time *t* and the information in two countries *c*_1_ and *c*_2_ including locations, GDP per capita and a series of indexes reflecting the government policy of *c*_1_ and *c*_2_. These indexes include the stringency index, the containment and health index, and the vaccination policy index [12]. We denoted the neural network as *f*_*θ*_, where *θ* are the corresponding trainable parameters in the hidden layers of the neural network. Here the neural network we adopt have five hidden layers and there are 10 neurons in each hidden layer and we use Relu function as the activation function in each layer.

We trained the neural network by the available data in the movement map provided by Facebook [7] and the corresponding flight data [6]. The proportion of aircraft seats occupied is calculated by:

(1) $$\begin{array}{c} \kappa_{c_{1},c_{2},t}=\frac{\Psi_{t}^{c_{1},c_{2}}}{r^{c_{1}}\Gamma_{t}^{c_{1},c_{2}}}, \end{array}$$

where Ψ_*t*_^*c*_1_,*c*_2_^ is the number of Facebook users traveling from country *c*_1_ to country *c*_2_ at time point *t* and *r*^*c*1^ is the proportion of population using Facebook in country *c*_1_. Γ_*t*_^*c*_1_,*c*_2_^ is the number of available flight seats from country *c*_1_ to country *c*_2_ at time point *t*.

**Figure 2:**
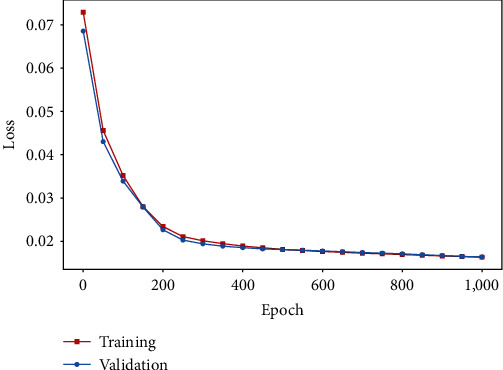
The training curve of the mean squared error (MSE) loss. The red curve represents the MSE loss between the real proportions and the predicted proportions for the training set in each epoch of training, while the blue curve represents the MSE loss between the real proportions and the predicted proportions for the validation set in each epoch of training.

We divided the entire dataset into a training set and a validation set using an 80 : 20 ratio. The neural network is optimized on the training set by Adam, a gradient-based optimizer, iteratively [18]. After the training is concluded, we can exploit the neural network to calculate the proportion of occupied seats on flights between any two countries that are occupied at time point *t*. We further use the validation set composed of known movement data to validate our model. We calculate the real proportions of aircraft seats occupied from the data in the validation set and use our trained neural network to predict the proportions of aircraft seats occupied for the validation set. We show the training curve of the mean squared error (MSE) loss between the real proportions and the predicted proportions for the training set and the validation set in Figure 2. We calculate the *R*^2^ score between the real proportions and the predicted proportions, which is 0.67. In previous work bu Bai et al. [5], the authors just exploited a fixed proportion of aircraft seats occupied while the *R*^2^ score between the real proportions and the fixed proportion is 0.11. Our results demonstrate that the neural network approach is capable of generating more precise predictions compared to the previous method.

Based on the predicted proportion of aircraft seats occupied, we estimate the movement population data *Ω*_*t*_^*c*^ from Western Africa to other non-African countries as given by

(2) $$\begin{array}{c} \Omega_{t}^{c}=\kappa_{\text{WA},c,t}\Gamma_{t}^{c}, \end{array}$$

where *κ*_WA,*c*,*t*_ represents the proportion of aircraft seats occupied as predicted by the neural network and Γ_*t*_^*c*^ is the count of seats on flights from Western Africa to destination *c*.

### 2.3. Risk of Importing Omicron BQ.1 through Infected Travelers from Western Africa

To evaluate the likelihood of introducing the BQ.1 subvariants from Western Africa, we initially computed the prevalence of asymptomatic and presymptomatic cases, based on the daily confirmed SARS-CoV-2 cases across the 17 countries described in the above data part. Note that we assumed symptomatic infected people are not allowed to travel across countries. Then we used movement data estimated above to estimate the importation risk of Omicron BQ.1 from Western Africa to non-African countries.

The previous study related to Omicron variant shows that 74.5% of infected cases of Omicron variant will develop symptom [13], and we denote such probability as *p*_sym_. Another study on the duration of the incubation period highlighted that interval between infection and symptom onset in symptomatic cases for Omicron variant has shortened to 3 days [15]. Marqez et al. [16] reported that the infectious period for asymptomatic and symptomatic cases lasts approximately 12 days and 7 days, respectively. Let *dI*_*t*_^WA^ denotes the number of new cases in Western Africa at time point *t* and *ω*_*t*_ denotes the proportion of the Omicron BQ.1 subvariants among new COVID-19 cases at a given time point *t*. The number of new asymptomatic and symptomatic cases in Western Africa at time point *t* is represented by *dI*_sym,*t*_^WA^ and *dI*_asym,*t*_^WA^, respectively, and they are estimated by

(3) $$\begin{array}{c} dI_{\text{sym},t}^{\text{WA}}=\omega_{t}p_{\text{sym}}dI_{t}^{\text{WA}}, \end{array}$$

(4) $$\begin{array}{c} dI_{\text{asym},t}^{\text{WA}}=\omega_{t}\left(1-p_{\text{sym}}\right)dI_{t}^{\text{WA}}, \end{array}$$

where *ω*_*t*_ denotes the percentage of the Omicron BQ.1 among new cases at time point *t*. Here we assumed the prevalence of BQ.1 in Western Africa is close to that in Nigeria which is identified as the origin of Omicron BQ.1 [2].

The number of new presymptomatic infections *dI*_presym,*t*_^WA^, the total number of presymptomatic infections and the total number of asymptomatic infections are estimated, respectively, by

(5) $$\begin{array}{c} dI_{\text{presym},t}^{\text{WA}}=dI_{\text{sym},t+D_{\text{presym}}}^{\text{WA}}, \end{array}$$

(6) $$\begin{array}{c} I_{\text{asym},t}^{\text{WA}}=\sum_{i=t-D_{\text{inf},a}}^{t-1}dI_{\text{asym},t}^{\text{WA}}, \end{array}$$

(7) $$\begin{array}{c} I_{\text{presym},t}^{\text{WA}}=\sum_{i=t-D_{\text{presym}}}^{t-1}dI_{\text{presym},t}^{\text{WA}}. \end{array}$$

Next we estimated the prevalence of asymptomatic and presymptomatic cases of Omicron BQ.1 subvariants in 17 countries we considered in the Western Africa by:

(8) $$\begin{array}{c} \xi_{t}^{\text{WA}}=\frac{I_{\text{asym},t}^{\text{WA}}+I_{\text{presym},t}^{\text{WA}}}{N_{\text{WA}}}, \end{array}$$

where *N*_WA_ is the total population of 17 countries we studied in the Western Africa.

Assuming that the proportion of infected travelers from Western Africa was the same as the overall prevalence of asymptomatic and presymptomatic cases of the Omicron BQ.1 variant at time point *t*. We approximated the rate of introducing cases from Western Africa to country *c* on time point *t* using

(9) $$\begin{array}{c} \gamma_{t}^{c}=\xi_{t}^{\text{WA}}\cdot \Omega_{t}^{c}, \end{array}$$

where *Ω*_*t*_^*c*^ denotes the number of tourists from Western Africa to country *c* at time point *t* is estimated by the method introduced above.

According to previous study by Wang and Wu [19], Wu et al. [20], and Tomba and Wallimba [21], we assumed that the introduction of cases of Omicron BQ.1 from Western Africa to other non-African countries follows a Poisson process, we calculated the likelihood of Western Africa exporting one or more Omicron BQ.1 subvariants to each country, denoted by *c*, by time point *t*, as follows:

(10) $$\begin{array}{c} \left(1-\text{exp}\left(-\sum_{i=t_{0}}^{t}\gamma_{i}^{c}\right)\right)/c_{0}, \end{array}$$

where *t*_0_ is the starting time and *c*_0_ is the scaling factor.

## 3. Results and Discussion

In this work, first, we developed a deep neural network model to predict the proportion of seats occupied on flights from the departure country to the destination country in the study period (from July 10 to September 30, 2022) based on the information in these two countries, such as locations, GDP per capita, and a series of indexes reflecting the government policy [12]. We assumed such information is associated with the movement population between these two countries. Combining with the flight data, we further estimated the number of population movements between two countries from July 10 to September 30, 2022, which enables us to fill in the missing values in the movement data offered by Facebook. Next, we estimated the probability of the introduction of Omicron BQ.1 to other countries from Western Africa based on the population mobility data estimated from Western Africa to non-African countries and COVID-19 case reports in the Western Africa.

**Figure 3:**
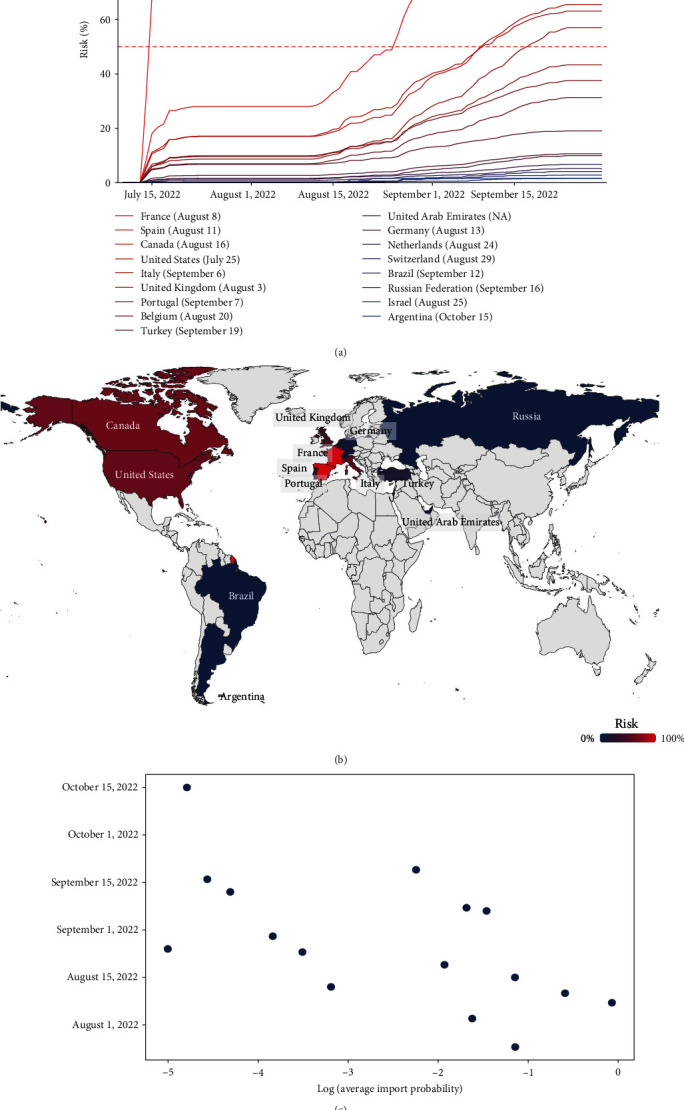
Estimated risks for importing Omicron BQ.1 from Western Africa to 17 countriesoutside Africa before 1 October 2022. (a) The probability of ≥1 Omicron BQ.1 infectedcase being imported to a target country from Western Africa by the date is indicated on the *x*-axis; The introduction probability of 50% is represented by the red dashed horizontal line;dates in parentheses are the timing of first BQ.1 documented in GISAID; (b) Probability of ≥1 infected case with the omicron BQ.1 importation from Western Africa before 1 October 1, 2022; countries in gray were not studied in our analysis because thecorresponding flight data from Western Africa to these countries were too few to make theestimation. (c) The logarithm of the average import probability for each country isrepresented on the *x*-axis. The figure illustrates that a higher import probability usuallycorresponds to an earlier arrival date of the infected cases.

The estimated importation risks varied by country and time. The highest risk was in France and Spain during the study period. The importation risk of France elevated rapidly from July 10, 2022 and the chance that France would receive one imported case from the Western Africa was at least 50% by July 13, 2022 and reached 100% by July 23, 2022 (Figure 3(a)). Notably, the probability of importation in Spain increased promptly after August 22, 2022, and the chance that Spain would receive one imported case was at least 50% by August 26, 2022 and reached 100% by September 4, 2022 (Figure 3(a)). Canada and the United States exceeded the 50% risk threshold on September 7, 2022 while Italy exceeded it on September 17, 2022. We estimated that eight countries outside Africa would have a greater than 30% probability of receiving one imported case by the end of September, 2022. The risk also existed for nine other non-African countries, including Turkey, the United Arab Emirates, and Germany (Figure 3(a)). We further projected the probability of importation into the world map (Figure 3(b)). The results indicate that Western Europe and northern American countries faced higher importation risk than the non-African countries in other countries, which might arise from many direct flights from Western Africa to these two countries. Furthermore, we have included a visualization of the logarithmic dependence of arrival time on import probability (Figure 3(c)). Following a similar methodology as by Brockmann and Helbing [22] and Gautreau et al. [23], we examined how the predicted import probability influences the arrival time of infected cases, as depicted in the log(*x*)-*y* plot. The figure reveals that a higher import probability typically corresponds to an earlier arrival date of the infected cases. This visualization provides valuable insights into the relationship between import probability and the timing of potential outbreaks.

Based on the above results, we found that non-African countries with numerous flights from Western African countries, such as France and Spain, are at a higher risk of harboring Omicron BQ.1. We observed that many cases were found in the countries with lower estimated risk such as the United States and the United Kingdom [2], which may be explained by their accurate and robust genomic surveillance program.

Our estimates were based on the epidemiological and direct flight assumptions by Bai et al. [5]. These assumptions may under/overestimate the importation risk of the target location. For example, as mentioned by Bai et al. [5], estimating population mobility data for direct flights and ignoring indirect flights may underestimate the risk of exports, especially if some infected carriers travel on the indirect flights.

## 4. Conclusions

In this work, we proposed a model based on deep neural network to estimate the importation risk of the Omicron BQ.1 variant. Our approach is able to yield accurate risk estimation from incomplete mobility data and shed light on how to exploit deep learning techniques to assist the development of public health policy. In the future, we will extend our approach to other types of viruses and provide a platform for policymakers to keep informed about the risks of importation.

## Figures and Tables

**Table 1:** Parameters, descriptions, and values.

Parameter	Description	Values
*N*_WA_	Population of Western Africa	466.62 million (2022) [8]
*N*_*c*_	Population of country *c*	Population estimates (2022) [8]
*r* ^*c*^	Proportion of population using Facebook in country *c*	Estimated Facebook users by country (2022) [9]
Ψ_*t*_^*c*_1_,*c*_2_^	Number of Facebook users traveling from country *c*_1_ to country *c*_2_ at time point *t*	Movement data of Facebook users [7]
Γ_*t*_^*c*_1_,*c*_2_^	Number of available flight seats from country *c*_1_ to country *c*_2_ at time point *t*	Flight data [6]
(*la*^*c*^, *lo*^*c*^)	Location of country *c*	Latitude and Longitude [10]
GDP^*c*^	Economic development level of country *c*	GDP per capita [11]
SI^*c*^	A combined metric derived from nine response indicators in country *c*	Daily stringency index [12]
CHI^*c*^	A composite measure of thirteen of the response metrics in country *c*	Daily containment and health index [12]
VPI^*c*^	Policies on the availability of vaccinations in country *c*	Daily vaccination policy index [12]
*Ω*_*t*_^*c*^	Number of travelers from Western Africa to country *c* at time point *t*	Estimated
Γ_*t*_^*c*^	Number of available flight seats from Western Africa to country *c* at time point *t*	Estimated
*κ*_*c*_1_,*c*_2_,*t*_	Proportion of available seats on flights from country *c*_1_ to country *c*_2_ that are occupied at time point *t*	Estimated
*p*_sym_	Proportion of infections exhibiting symptoms	74.5% [13]
*dI*_*t*_^WA^	Number of new cases in Western Africa at time point *t*	Daily new infections [14]
*ξ*_*t*_^WA^	Percentage of asymptomatic and pre-symptomatic cases of the variant among the population of Western Africa at time point *t*	Estimated
*γ*_*t*_^*c*^	Rate of introductions of the variant from Western Africa to country *c* at time point *t*	Estimated
*ω*_*t*_	Proportion of the Omicron BQ.1 subvariants among new cases at time point *t*	Proportion of Omicron BQ.1 subvariants cases in Nigeria [2]
*D*_presym_	Estimated interval for the onset of symptoms following infection in symptomatic cases	3 Days [15]
*D*_inf,s_	Estimated interval between infection and recovery, for symptomatic infections	12 Days [16]
*D*_inf,a_	Estimated interval between infection and recovery, for asymptomatic infections	7 Days [16]

GDP, gross domestic product.

## Data Availability

The mobility data and the flight data supporting our study are from previously reported studies and datasets, which have been cited. The processed data are available from the corresponding author upon request.
